# Ranking Trust Factors Affecting Risk Perception in Illicit Drug Purchase on the Darknet: A Large-Scale Survey Study in Hungary

**DOI:** 10.1007/s10610-023-09545-x

**Published:** 2023-05-22

**Authors:** Tibor Kiss, Ákos Szigeti

**Affiliations:** 1 Department of Criminology at University of Public Service, 2 Ludovika Tér, 1083 Budapest, Hungary; 2 Doctoral School of Law Enforcement, University of Public Service, 2 Ludovika Tér, 1083 Budapest, Hungary

**Keywords:** Trust, Darknet, Drug trafficking, Crime prevention

## Abstract

The process of illicit drug trafficking on darknet markets is highly affected by various trust factors. Although the factors potentially affecting customers’ risk perception can be identified based on previous research, cyber criminology has not produced empirical research ranking the importance of the specific factors. This study was designed to fill this gap by developing a tool that measures the importance of the various trust factors. To test out the measurement tool, a large-scale survey with projective situational questions was conducted among university students in Hungary. The sample (*n* = 5481) was compiled to include potential darknet market customers, respondents with above-average computer skills needed to access the darknet, and taking into account that university students are a group of society particularly exposed to drug consumption. The end product of this research is a trust matrix ranking the factors affecting illicit drug purchases on darknet markets. Among the factors, the survey’s target group ranked reliable and undamaged delivery of goods and the reliability of vendors as the most important. The measurement tool developed in this research will facilitate further criminological research on vendor reputation. Its findings also point to the need for further research on delivery providers and predict that influencing the delivery-related risk perception of potential customers could effectively reduce demand.

## Introduction 

According to the United Nations Office on Drugs and Crime, more than 60% of all trading on the darknet consists of illegal drug trafficking (United Nations Office on Drugs and Crime, 2021). While traffic to darknet markets has been growing steadily over the past decade, this growth has stalled recently (European Monitoring Centre for Drugs & Drug Addiction, 2022), primarily due to delivery issues caused by the COVID-19 pandemic lockdowns that have disrupted the trust-based relationship between vendors and customers (Bergeron et al., 2022a) . Therefore, traffic trends in darknet markets seem to reflect changes in the importance of certain elements of horizontal trust between vendors and customers. At the same time, the online drug trade does not appear to be going away but is moving from the darknet to more conventional encrypted messaging applications (van der Sanden et al., 2022), building on the vendor-customer relationships that were previously established either online or offline.

The horizontal trust that connects vendors and customers in popular online drug markets has evolved in recent decades because of information communication technology and global networking in legal online trade. Its novelty lies primarily in the fact that customers and vendors trust each other without meeting in person before closing a deal (Botsman, 2017). Empirical research on legal online markets has shown various factors affecting customers’ trust and propensity to buy (Al-Dwairi, 2013; Karimov & Brengman, 2014; Thaw et al., 2009). Even though the importance of these trust factors is constantly changing, there are particularly crucial aspects that can affect customers’ decisions, and most of these are grouped around risk perception (Ilmudeen, 2019). The reputation dynamics in the vendor-customer relationships on darknet markets have been very similar to surface web markets (Espinosa, 2019; Janetos & Tilly, 2017; Przepiorka et al., 2017). However, while some factors of horizontal trust are equally important in legal and illegal trade (for example, reliability of vendors, payment options, or quality of goods) (Spagnoletti et al., 2022), others are not as prominent in darknet purchasing (e.g., legal and data protection guidelines, guarantees of buyers’ rights, public relations, and registration options) and those which are more beneficial in illegal trade (e.g., anonymity, secret operation) than in legal online shopping. Since the closure of the Silk Road darknet market in 2013, horizontal trust has encouraged the development of self-regulating communities between suppliers and customers that can operate without external control in illicit trade (Bancroft, 2019; Martin, 2014; Martin et al., 2020). The various trust factors can reduce the risks associated with darknet drug purchases and motivate new customers who previously would not have bought illegal substances in a traditional environment (offline) due to the high risks (Holt et al., 2016; Oksanen et al., 2020; Pergolizzi et al., 2017).

This research study was designed to develop and test a trust metric that can provide a detailed understanding of trust factors affecting the risk perception of potential customers purchasing illegal drugs on the darknet. We developed a quantitative questionnaire with 21 items indicating trust, based on previous research on the role of trust in surface web and darknet (drug) trade. To test out this measurement tool, we selected a population less likely to purchase illegal drugs offline but more willing to do it online, anonymously, and privately, while its purchase intention is still likely to be reversible. The assessment of this trust metric enables ranking the trust factors and their comparison by relevant independent variables. Findings contribute to strategically planning demand reduction projects and interventions based on a deeper understanding of customers’ risk perception.

## Trust Factors and Hypotheses

In the quantitative questionnaire, we applied a trust scale of 21 items developed based on previous research results on legal and illegal online purchase transactions. These empirical studies focused on the trust factors in online purchasing, which consumers considered indispensable for safe online shopping. To facilitate the application, we have created seven categories and classified the trust factors into these categories (see Table 1).

**Table 1:** List of trust categories and factors developed by the authors

Trust categories	Trust factors
Individual and community	Vendor contact and helpfulness (TF 1)
	Vendor reliability (TF 2)
	Web store popularity and positive feedback on the internet (TF 3)
	Store recommended by acquaintances or friends (TF 4)
Technology and network	Ease of navigating the web store page (TF 5)
	Stable internet connection while shopping (TF 6)
Data protection, privacy, and legal information	Protection of personal and purchase data (TF 7)
	Information on the rights and obligations of buyers and vendors (TF 8)
	Option of registering, or not, for browsing and purchases (TF 9)
Payment transaction	Accessible, fast, and easy payment transaction (TF 10)
	Online payment option (TF 11)
	Traditional offline payment option (TF 12)
	Payment option with cryptocurrency (TF 13)
	Instant confirmation of your payment (TF 14)
Products and services	Wide range of products and services, good quality and value for the price (TF 15)
	Vendors or manufacturers of goods or services guarantee (full refund or escrow) (TF 16)
	Adequate information about prices, goods, and services offered (TF 17)
Delivery services	Reliable delivery of goods (TF 18)
Anonymity and encryption	Opportunity to buy in a hidden and untraceable way (TF 19)
	My purchase information should be deleted immediately (TF 20)
	Vendor should not know my real data (TF 21)

The category of individual and community factors includes items related to the popularity, recommendation, and reliability of the seller and the platform. Customers’ trust and propensity to buy depend on the perceived reliability of the online retailer (Thaw et al., 2009) and acceptance of the given web market (Karimov & Brengman, 2014). Similarly to surface web markets, most darknet markets create reputation systems in which customers share their experiences with other web market visitors about the product and its quality, the vendor’s helpfulness, the structure and selection of the market, and the delivery of the products (Holt et al., 2016; Janetos & Tilly, 2017). The reputation system consists of customer ratings, reviews of products or services purchased, number of visitors, and number of purchases. Furthermore, a good reputation or positive perception disseminated by word of mouth can be even more effective than the reputation system (Duxbury & Haynie, 2018; Przepiorka et al., 2017).

The technology and network category includes trust factors regarding the user experience on websites and the smoothness of internet connections. The success of web markets is greatly facilitated by their well-designed and well-structured interface, since the persuasion of customers begins when they first visit the website. The structure and manageability of the website (Al-Dwairi, 2013) and the performance and stability of the market interface (Karimov & Brengman, 2014) both affect the purchasing process on surface web markets. In addition to easy navigation, this category also includes a stable internet connection, which mostly means network access to the website and uninterrupted shopping operations, i.e., the connection between the customer and the web market should not be interrupted during the payment transaction (Ilmudeen, 2019).

Data protection, privacy, and legal information form another necessary category of trust factors. Data protection means the management, storage, and protection of purchase-related transaction data (e.g., payment and invoicing data) and the customer’s personal information (personal identification data, name, address, telephone number, and e-mail address provided by the customer) (Ilmudeen, 2019; Thaw et al., 2009). Registration data, legal documents, and guidelines indicating buyer and seller rights and obligations are also listed here (Karimov & Brengman, 2014). Providing information on privacy protection is essential for legally operating web markets. For their illegal counterparts, confidential data treatment is key to survival, as data protection is fundamental in reducing the risk of being caught by the authorities (Thaw et al., 2009). Data protection obligations also apply to the vendor in sending product advertisements and informing potential customers. The recording and subsequent use of email addresses are subject to regulations, thus vendors must display the relevant information (Al-Dwairi, 2013).

The payment transaction is an essential element of online shopping for several reasons. When using an on-demand interface, the speed and simplicity of the payment transaction can make or break a sale (Ilmudeen, 2019). On most legal web markets, offline and online payment alternatives are available to customers. Offline payment is a confidence-building factor for those who do not dare or are unable to pay online and those buying higher-value products. Meanwhile, online payment is widely popular and is usually provided by reputable financial institutions, which can have a confidence-boosting role. Although an option to pay in cryptocurrency is still uncommon in legal e-commerce, there are obvious advantages to paying in cryptocurrency on illegal markets, as this kind of payment path is usually made untraceable by cryptocurrency mixers (Norbutas et al., 2020). However, cryptocurrencies are traceable to varying degrees; for example, Bitcoin is easier to trace than Monero (Bahamazava & Nanda, 2022). Finally, immediate feedback on the payment outcome adds an ultimate confirmation to the customer regarding the payment.

The products and services category includes trust factors regarding products. Many trust-building factors related to products can be highlighted. Among these, trust in the quality of products and their perceived value for money are important elements, in addition to the accurate description and visual presentation of the products, which are as important as optimal pricing (Al-Dwairi, 2013; Karimov & Brengman, 2014). Legal web markets often use the price comparison function to allow customers to compare prices with other online markets. Displaying discount prices or waiving shipping fees can act as a marketing ploy. Another trust-building factor is offering a wide range of products so that customers can choose between, for example, goods of lower or higher quality or from various manufacturers. The importance of weight, value, potency, purity, and price of drug products was highlighted by previous darknet studies as well (Bancroft & Reid, 2016; Munksgaard et al., 2022). In addition, assuring the customer that the vendor will refund the value of defective or unsuitable goods or that the vendor will replace it with an intact product is a guarantee which exists not only in legal web markets but also in most darknet markets (Bancroft & Reid, 2016).

The conditions for delivering goods have been placed in the delivery services category. In the majority of web markets, there are several alternatives for delivery. In addition to choosing between home delivery or delivery to a pick-up point, consumers have the option to plan the time and place of the receipt of the goods, including cancellation and rescheduling. All of these together make up the trust factor in transportation. This trust also extends to whether the person who ships and delivers the goods reaches the destination at all, whether the package disappears in the course of delivery, or whether the goods are stolen or lost before they reach the recipient (Martin, 2014; Martin et al., 2020; Özçiçek Dölekoğlu Celile, 2019). Such customer doubts may arise especially in the case of drugs or illegal pharmaceuticals ordered from darknet markets, where the products can be seized by the authorities or stolen by someone, and therefore, no legal guarantee can be enforced by the customer (Oksanen et al., 2020; Przepiorka et al., 2017). The importance of the integrity of the goods cannot be overlooked either, especially for fundamentally fragile or highly valued products.

Finally, the category of anonymity and encryption refers to the untraceability of online shopping and the unidentifiability of the customer. In legal commerce, this is only relevant in terms of storing and managing data related to the purchase and the customer (Ilmudeen, 2019). Some online shoppers are only willing to provide their personal data at the point when they order the products but refuse to register and ask the seller to delete all information about them. Stealth becomes more significant in illegal than in legal commercial transactions, where invisibility can prevent exposure or embarrassment (Aldridge & Askew, 2017). This demand can occur not only when ordering illegal products but also when buying sensitive products, such as health or sexual products. We have listed three factors under the anonymity and encryption category. The first is the encryption of the entire purchase process, i.e., the immediate deletion of online traces of the customer. The second is the prohibition of retaining purchase data, which embodies the demand expressed by the customer towards the vendor or the web market’s operator (Dordal, 2018; Espinosa, 2019). The third is complete anonymity, which also extends to the actor from whom the customer orders the product. This trust factor is based on mistrust, which becomes essential in illegal trade processes (Przepiorka et al., 2017).

Two hypotheses were formulated regarding ranking the above-presented factors, while an additional hypothesis was formulated about a decision to purchase the same substance online or offline.

- Hypothesis 1 (H1): All trust factors are considered necessary in legal and illegal online purchasing situations.

Testing the first hypothesis highlights whether there are similarities or differences in the process of legal and illegal online shopping. Based on the answers to the related questions, we can highlight the most important trust factors in illegal trade.

- Hypothesis 2 (H2): The factors related to anonymity and encryption are considered more important in illegal online purchasing situations compared to legal online purchasing.

Anonymity and encryption are the main factors contributing to the operation of drug trafficking on darknet markets, according to a number of previous studies. Examining the differences between the role of the related factors in legal and illegal purchases can help us understand the operation of illegal drug purchases.

- Hypothesis 3 (H3): If it is not possible to purchase the drug in a legitimate commercial way, but it is possible to purchase it illegally both offline and online, users would prefer to order the substance on an online platform.

By examining the third hypothesis, we will get to know the respondents’ attitudes toward the opportunities available online, and we will be able to tell what proportion of customers would choose to purchase online or offline.

## Methods

The current study aims to rank the importance of trust factors affecting the risk perception of potential darknet market customers through an online survey to lay the foundations for crime prevention strategies. While prevention should target potential customers, survey research involving the users of darknet markets usually ends up being small-scale (Bergeron et al., 2022b; Karden & Strizek, 2022). To overcome this issue, we selected a population whose members have characteristics that help them place themselves in fictitious purchasing situations on the darknet. This projective (third-person) technique can help when respondents do not want to reveal their real attitudes about something (Kumar et al., 2018) and has already been applied in cyber criminology (Parti et al., 2018). This approach lets us compile a sample that is large enough to perform multivariate statistical analysis to explore trust factors behind purchasing decisions of (potential) DNM customers.

The survey was designed to be disseminated among Hungarian university students for the following reasons: (1) they have the above-average computer skills needed for accessing the darknet; (2) they are one of the groups in society that are the most exposed to drug abuse; (3) there are subgroups among them who have above-average knowledge on the technical, legal, or medical aspects of online drug trafficking, providing an opportunity for further comparative analysis; and (4) they could be directly contacted through the online education administration systems and internal email services of Hungarian universities. While this sample selection limits our results to a social group with a given age and education, it also enables the planning of targeted crime prevention strategies based on primary data collected directly from the selected population.

The research plan was approved by the Cybersecurity Research Institute of the Eötvös József Research Center at the University of Public Service, Hungary. The survey was conducted in the well-protected LimeSurvey data collection, analysis, and evaluation system. Participation in the online survey was completely anonymous and voluntary. The questionnaire was self-administered by respondents, and each of its questions could be skipped; therefore, the system also recorded partially completed questionnaires.

External researchers were involved in the process of survey design in two stages: (1) an internal workshop was organized at the indicator development stage, and (2) the draft of the survey was presented and discussed at an international cybercrime workshop. The structured questionnaire mainly contained closed-ended questions, with only a few other open-ended options. In the first block of questions, we asked the respondents about their internet habits, general attitudes (e.g., trust, zero tolerance for drug use), and knowledge about illegal drugs. Situations adapted from real-life stories were added to the following five question blocks, to which the respondents could respond by evaluating the importance of trust factors listed under the situations (see Table 2).

**Table 2:** Abridged descriptions of illicit drug purchasing situations used in the questionnaire

Titles of the situations	Shortened descriptions of the situations
Ferenc is a single man: Purchasing medicine for his injured mother (IS1)	Ferenc is a middle-aged man whose mother has had several joint surgeries due to a severe accident and suffers unbearable pain. The legal medicines are ineffective, so Ferenc, at the suggestion of a friend, turns to darknet markets where he tries to buy illegal medicine containing codeine
Andrea is a medical student: Purchasing drugs to increase her learning capacity (IS2)	Andrea is an overburdened medical student with too many examinations in the last semester of university. Because of this, she cannot study enough until the exam. Coffee and other caffeinated legal substances are no longer as effective. Her friends suggested that she get some amphetamine pills to boost her performance so she could study more in the short preparation time and pass her exams. Andrea decided to buy amphetamine pills on the darknet
Karolina is a mother: Purchasing life-saving medicines for her son (IS3)	Karolina is a mother in her 30 s. She lives with her husband and three children in a small town. One of her children suffers from a fatal and chronic disease, but she cannot afford the pharmaceutical treatment. Karolina even organized a fundraising drive to raise money, but only half of the money was raised. Because of this, she has decided to buy the medicine on the dark web at half price
Gergő is a careerist: Purchasing drugs to increase his working capacity (IS4)	Gergő is a 25-year-old man who is a manager at a large international company. He works incredibly hard but receives a high salary, which allows him to live a luxurious life. The company’s expectations are increasing, and Gergő cannot handle the load alone. In order to increase his performance, he sometimes consumes cocaine. Because of the risks involved with buying it in person, he decided to order the cocaine on the darknet market
Géza is a party-loving young man: Purchasing drugs for a party (IS5)	Géza is a young man who goes to Budapest once every month to have fun with his foreign friends. In these events, they regularly consume marijuana wrapped in cigarette paper. Until now, his friends provided the marijuana every time, but now it is his turn. Géza decided to order the drug on the darknet

Respondents were asked to decide what they would do in these situations by selecting action options. These projective questions allowed us to learn about what decisions respondents might make in critical situations and their willingness to act. The last block of questions was about the respondents’ demographic characteristics.

An invitation to participate in the online survey was sent out to 63 Hungarian higher education institutions registered on the official online portal of the Hungarian Office of Education. Of these institutions, 28 agreed to participate and disseminated the questionnaire through their internal email systems. The survey was open for completion from September 1 to November 15, 2020. The questionnaire was opened 8051 times during this period, and 5481 responses were recorded. To test Hypotheses 1 and 2, we used only the responses which ranked all trust factors in all situations (*n* = 1162). To test Hypothesis 3, we were able to use a larger amount of answers in each situation, the number of which decreased as the situations appeared in the questionnaire.

Data cleaning and data analysis were made exclusively in IBM SPSS Statistics. The trust matrix was created by ordering the means of the importance of each trust factor. Due to the lack of a normal distribution of the sample, we performed non-parametric tests in addition to presenting descriptive statistics.

## Results

### Ranking the Importance of Trust Factors

Considering that there were only minor differences in the means of the importance of the trust factors when comparing the five illegal purchasing situations, we tested the internal consistency of each factor across the situations (see Table in Appendix). The results of Cronbach’s Alpha tests indicated high internal consistency (> 0.8) for each item; thus, index variables were created to summarize the importance of each trust factor in illegal purchases.

When the results of the new index variable regarding illegal purchases were compared to legal purchases, it partially confirmed Hypothesis 1. While most factors were considered at least rather important (≥ 2.5) in both legal and illegal purchasing situations, there were some differences between the two cases (see Table 3). Specifically, the factor regarding registration (TF 9) and the option to pay by cryptocurrency (TF 13) were ranked as not important (< 2.5) in both situations. While the factors of purchasing in a hidden and untraceable way (TF 19) and of customers’ details being kept hidden from the vendor (TF 21) were considered not important (< 2.5) in the legal purchasing situation, these two factors were considered very important (≥ 3.5) in the illegal situations.

**Table 3:** Trust matrix: mean values of the importance of trust factors in legal purchasing situation (LS), in illegal purchasing situations (index of the five situations, IS1-5), and their rankings based on Wilcoxon signed rank test asymptotic significance (two-tailed), *n* = 1162

	LS	IS1-5	Ranked higher	Sig
Vendor contact and helpfulness (TF 1)	3.56	3.44	LS	< 0.001
Vendor reliability (TF 2)	3.89	3.79	LS	< 0.001
Web store popularity and positive feedback on the internet (TF 3)	3.21	3.18	LS	< 0.001
Store recommended by acquaintances or friends (TF 4)	2.63	3.23	NS	0.696
Ease of navigating the web store page (TF 5)	3.51	3.25	IS	< 0.001
Stable internet connection while shopping (TF 6)	3.46	3.52	LS	< 0.001
Protection of personal and purchase data (TF 7)	3.79	3.7	LS	< 0.001
Information on the rights and obligations of buyers and vendors (TF 8)	3.28	3.02	LS	< 0.001
Option of registering, or not, for browsing and purchases (TF 9)	2.36	1.71	IS	< 0.001
Accessible, fast, and easy payment transaction (TF 10)	3.57	3.6	IS	0.003
Online payment option (TF 11)	3.3	3.09	LS	< 0.001
Traditional offline payment option (TF 12)	2.84	2.84	NS	0.729
Payment option with cryptocurrency (TF 13)	1.66	2.36	IS	< 0.001
Instant confirmation of your payment (TF 14)	3.82	3.4	LS	< 0.001
Wide range of products and services, good quality and value for the price (TF 15)	3.75	3.52	LS	< 0.001
Vendors or manufacturers of goods or services guarantee (full refund or escrow) (TF 16)	3.56	3.2	LS	< 0.001
Adequate information about prices, goods, and services offered (TF 17)	3.81	3.65	LS	< 0.001
Reliable delivery of goods (TF 18)	3.95	3.85	LS	< 0.001
Opportunity to buy in a hidden and untraceable way (TF 19)	1.92	3.54	IS	< 0.001
My purchase information should be deleted immediately (TF 20)	2.71	3.7	IS	< 0.001
Vendor should not know my real data (TF 21)	2.36	3.6	IS	< 0.001

These results predicted the confirmation of Hypothesis 2 about the higher ranking of anonymity and encryption-related factors in an illegal purchasing situation. Indeed, in addition to the factors mentioned above on untraceability and hidden details, the factor about deleting purchase information immediately (TF 20) was also ranked as significantly more important in the index of illegal situations based on the results of Wilcoxon signed rank tests (*p* < 0.001).

In addition to testing Hypotheses 1 and 2, it should be highlighted that reliable delivery of goods (TF 18) and vendor reliability (TF 2) were ranked at the top. In contrast, the option of registering (TF 9) and payment with cryptocurrency (TF 13) were ranked at the bottom of the list of the factors examined in the illegal purchasing situations.

### Favoring the Darknet to Purchase Drugs

To test Hypothesis 1, the choice of an in-person or online purchase was analyzed in each situation (see Table 4). The differences were measured by chi-square goodness-of-fit tests, which showed that respondents significantly preferred to use online dark web marketplaces to purchase drugs compared to in-person buying options. While the proportions of those who selected the online or the in-person option varied among the situations (see below), respondents preferred the online option in each situation. However, the difference was only significant in the first four situations (IS1, IS2, and IS3: *p* < 0.001; IS4: *p* = 0.043); the respondents were almost equally divided in the fifth situation, in which the difference was not significant (*p* = 0.553).

**Table 4:** Distribution of respondents who selected the online and in-person option to purchase drugs, broken down by scenarios, with chi-square goodness-of-fit tests

	IS1	IS2	IS3	IS4	IS5
I would rather order online	1707	1374	1196	784	697
I would rather buy in person	1266	968	592	706	675
n	2973	2342	1788	1490	1372
Asymp. Sig	< 0.001	< 0.001	< 0.001	0.043	0.553

Respondents who selected the offline option were asked to elaborate their answers in an additional optional, multiple-choice question with predefined choices (see Table 5). The majority of these respondents (36 to 42%, depending on the situation) would be afraid of being scammed or that the goods (drugs) would never arrive. Fewer respondents (31 to 38%) said that it was important for them to be there in person when choosing the goods and to see what they were buying. Finally, even fewer respondents (25 to 29%) said they did not know how to buy drugs online because they have never done that. There was one significant difference between the situations: in the fifth situation, a slightly higher percentage of respondents said that they would choose the offline option because they would prefer to purchase in person than the otherwise more popular answer about the fear of being scammed (38 compared to 36%).

**Table 5:** Distribution of respondents by reason of selecting the in-person option to purchase drugs

	IS1	IS2	IS3	IS4	IS5
I do not know how to purchase drugs online, I have never done that	27.4%	29.0%	25.2%	26.9%	26.8%
I would be afraid of being scammed and that the product would not arrive	41.9%	38.5%	42.3%	39.2%	35.5%
It is important for me to be there in person and see what I purchase	30.7%	32.5%	32.5%	33.9%	37.7%
n	1630	1281	840	970	932

In sum, Hypothesis 3 was confirmed: respondents preferred to purchase drugs online.

## Discussion and Recommendations

The target group of the research, composed of potential customers, ranked the reliable delivery of goods (TF 18) as the most important trust factor when buying illicit drugs on the darknet. This result is in line with the findings of a wide range of previous research, claiming that the highest risk of the online drug purchase process is in the delivery and receipt stage (Bancroft & Reid, 2016; Espinosa, 2019; Jardine, 2021; Lorenzo-Dus & Di Cristofaro, 2018). Although this survey study was not conducted among actual darknet market users, our findings and its extensive support by previous literature suggest that delivery issues indeed drive the recent stall in the volume of the darknet drug trade. In order to better understand the role of delivery providers, the list of trust factors measured in future survey studies could include more delivery-related factors measuring how speed, stealth, packaging, and delivery service providers contribute to the perception of reliable delivery (Szigeti et al., 2023). Furthermore, examining the postal and other delivery services, including packet inspection methods and delivery protocols, could facilitate the evidence-based re-evaluation of delivery regulations and could ultimately affect customers’ decisions. Ultimately, reforming the protocols of delivery service providers and implementing targeted risk awareness campaigns could reduce both the supply and demand on darknet markets by influencing customers’ and vendors’ delivery-related risk perception.

Respondents ranked vendor reliability (TF 2) as the second most important trust factor in illegal drug purchasing on the darknet. This factor is the foundation of reputation systems which are the strongest trust guarantees in illegal web markets and which allow customers to check vendors’ activities and details, including their number of transactions, ratings, reviews, and prices (Holt et al., 2016; Janetos & Tilly, 2017; Munksgaard & Tzanetakis, 2022; Norbutas et al., 2020). In addition, most of the respondents would prefer to purchase drugs online than in person. Both findings indicate that the perception of the reliability of markets and vendors determines potential customers’ decisions. The escrow system used in darknet markets provides an additional technical guarantee of payment security by depositing the price of the ordered product and releasing it upon receipt if both the vendor and the customer agree (Moeller, 2022). This factor was measured only as an element of guarantees, but its relevance would justify its examination as a separate trust factor if the survey would be conducted among actual darknet market users. Similarly, the emphasis on the number of transactions a given vendor completes in recent literature (Munksgaard et al., 2022) could justify adding a dedicated factor measuring its importance. Regarding policy implications, the findings suggest that emphasizing the risks that consumers are exposed to concerning theft, fraud, fake sites, and products could facilitate crime prevention. For example, warnings about scammers could directly influence the activity of vendors and customers on a given darknet market (Howell et al., 2022).

The fact that most of the respondents did not consider the registration option (TF 9) and payment with cryptocurrency (TF 13) to be essential trust factors contradicts the practice of darknet drug trade (Bahamazava & Nanda, 2022; Szigeti et al., 2023). This may be due to the composition of the sample since the technical elements of darknet purchasing are only learned by potential customers at the so-called informational accumulation stage of the process of the darknet drug trade (Jardine, 2021). Therefore, actual darknet market users who have the required technical knowledge would perhaps assess these factors differently. Furthermore, in line with research highlighting the importance of anonymity and encryption in the process of the darknet drug trade (Ilmudeen, 2019; Przepiorka et al., 2017), the related trust factors (TF 19, TF 20, and TF 21) were ranked as significantly more important in illegal purchasing situations in comparison to legal purchasing. The lack of technical knowledge and the assessment of other elements of anonymity and encryption suggest that risk perception related to data loss can be effectively influenced in the study’s target population. Some initiatives can effectively increase users’ perception of risks. An example is Operation Bayonet, in which law enforcement agencies took over an entire darknet market and obtained user data, thus effectively reducing the activity of vendors and customers (Bradley & Stringhini, 2019). Informing potential users that their identity is not completely hidden, even when purchasing on the darknet, may also impact their perception of risk.

The findings, criminal policy recommendations, and criminological research remarks presented above are summarized in Table 6.

**Table 6:** Key criminological research remarks and criminal policy recommendations by results and hypotheses

Hypotheses	Results	Criminological research remarks	Criminal policy recommendations
H1	Reliable delivery of the goods (TF 18) was ranked as the most important trust factor	Measuring the various elements of the perception of reliable delivery and examining delivery protocols	Re-evaluation of delivery protocols and influencing risk perception related to the delivery of products
H1	Vendor reliability (TF 2) was ranked as the second most important trust factor	Escrow service as a technical guarantee and the number of transactions completed by the vendor could be measured as separate trust factors in future studies conducted among actual darknet market users	Emphasizing the unreliability of illegal markets and vendors by targeted interventions and campaigns
H3	The dominance of the online purchase and a small group of skeptics		
H1	Registration option (TF 9) and paying by cryptocurrency (TF 13) were not considered necessary trust factors	The survey should also be conducted among actual darknet market users to understand the operation of the various trust factors in darknet purchasing	Emphasizing the risk of data loss through targeted interventions and risk awareness campaigns
H2	Factors related to anonymity and encryption (TF 19, TF 20, and TF 21) were ranked higher in illegal purchase		

While the above-mentioned examples of interventions effectively reduced the volume of the darknet drug trade, the policy recommendations presented above were designed to be implemented among potential customers. Since some users purchase on the darknet because they do not want to risk a personal meeting with dealers, darknet markets extend the range of potential users (Pergolizzi et al., 2017). While these users might be deterred from purchasing drugs, the effectiveness of influencing the risk perception of actual darknet market customers who may be addicted drug users is questionable. Furthermore, deterring drug users from the darknet could lead to them buying drugs on the streets, which could be more dangerous from a health perspective. Drugs on the darknet have a better reputation regarding cleanliness (Bancroft, 2017; Munksgaard et al., 2022), and some form of harm reduction also derives from the communities formed around darknet markets (Aldridge et al., 2018; Szigeti et al., 2023). Policy recommendations tailored to actual darknet market users should therefore be developed based on further research implemented in the communities of darknet markets. Interventions should then be integrated into a comprehensive drug policy framework that includes other elements, such as targeted harm reduction and treatment.

## Limitations

This study was designed to list trust factors potentially affecting illicit drug purchases and to test out their ranking by using a survey study based on a projective technique. Therefore, this research is limited by the fact that it could not build on the results of previous survey research. However, the results of this study could facilitate further research in replicating this projective methodology or applying the list of factors as a module of a questionnaire to be conducted among actual darknet market users.

The sample selection of this research was determined by the research concept, in which direct involvement and representativeness were preceded by special characteristics and skills of the research subjects and the analysis possibilities provided by the relatively large sample size. Therefore, the survey was not conducted among darknet market customers, and it is not representative for the country in which it was carried out. However, its results can provide evidence-based recommendations for interventions targeted at university students.

The majority of respondents only filled out the questionnaire in part, which can be explained by the length of the survey and its complex, repetitive situational questions. While it is unknown how those who completed the questionnaire differ from those who filled it out partially, shortening the questionnaire in future fielding is advised. The results of the internal consistency tests presented above suggest that reducing the number of situations could be a solution that would result in little data loss, especially if comparing the illegal situations is not among the aims of the given research.

Finally, despite its large size, the sample only allowed us to perform non-parametric tests due to the lack of normal distribution. The re-evaluation of the indicators applied in the questionnaire, taking statistical aspects into account, could help create a database suitable for running more robust parametric tests and developing models.

## Conclusions

The empirical research findings of this study have provided lessons that can be used in the design of future criminological research and the strategic planning of criminal policy. The main contribution of this research is the development of the trust matrix, which can enhance criminological research on how trust works in darknet transactions. Based on the results, the developed trust metric can be further extended and queried among actual darknet market users. The responses given by potential darknet market customers highlighted the role of delivery, vendor reliability, and anonymity-related trust factors in their risk perception. Based on these results, this study concluded that influencing the risk perception of potential darknet market customers regarding transaction and delivery could contribute to reducing the volume of the darknet drug trade. However, the effectiveness of the recommended regulatory measures, law enforcement interventions, and prevention methods among actual darknet market users is questionable and should be further examined by future research.

## Data Availability

The datasets generated by the survey research and analyzed in this study are available from the corresponding author upon reasonable request.

## Appendix

Mean values of the importance of trust factors in illegal purchasing situations (IS) and the results of the Cronbach’s Alpha (CA) internal consistency tests

**Table:**

	IS1	IS2	IS3	IS4	IS5	Cronbach’s Alpha of IS1-5
Vendor contact and helpfulness (TF 1)	3.38	3.40	3.65	3.36	3.42	0.89
Vendor reliability (TF 2)	3.83	3.79	3.86	3.74	3.75	0.85
Web store popularity and positive feedback on the internet (TF 3)	3.11	3.07	3.31	3.12	3.27	0.91
Store recommended by acquaintances or friends (TF 4)	3.23	3.24	3.20	3.11	3.35	0.90
Ease of navigating the web store page (TF 5)	3.18	3.21	3.37	3.17	3.30	0.92
Stable internet connection while shopping (TF 6)	3.54	3.52	3.53	3.52	3.49	0.94
Protection of personal and purchase data (TF 7)	3.74	3.72	3.70	3.68	3.66	0.89
Information on the rights and obligations of buyers and vendors (TF 8)	2.92	2.97	3.20	2.98	3.01	0.94
Option of registering, or not, for browsing and purchases (TF 9)	1.63	1.63	1.81	1.72	1.76	0.93
Accessible, fast, and easy payment transaction (TF 10)	3.58	3.59	3.61	3.62	3.60	0.90
Online payment option (TF 11)	2.95	3.06	3.18	3.11	3.14	0.94
Traditional offline payment option (TF 12)	2.83	2.81	2.85	2.84	2.88	0.94
Payment option with cryptocurrency (TF 13)	2.38	2.34	2.30	2.44	2.33	0.96
Instant confirmation of your payment (TF 14)	3.34	3.38	3.48	3.38	3.41	0.93
Wide range of products and services, good quality and value for the price (TF 15)	3.42	3.47	3.59	3.51	3.59	0.88
Vendors or manufacturers of goods or services guarantee (full refund or escrow) (TF 16)	3.13	3.10	3.43	3.15	3.18	0.92
Adequate information about prices, goods, and services offered (TF 17)	3.68	3.65	3.77	3.55	3.59	0.88
Reliable delivery of goods (TF 18)	3.83	3.84	3.89	3.85	3.83	0.87
Opportunity to buy in a hidden and untraceable way (TF 19)	3.51	3.55	3.44	3.63	3.59	0.92
My purchase information should be deleted immediately (TF 20)	3.73	3.71	3.60	3.75	3.70	0.90
Vendor should not know my real data (TF 21)	3.60	3.64	3.49	3.68	3.61	0.90
